# West Nile alternative open reading frame (N-NS4B/WARF4) is produced in infected West Nile Virus (WNV) cells and induces humoral response in WNV infected individuals

**DOI:** 10.1186/1743-422X-9-283

**Published:** 2012-11-22

**Authors:** Giovanni Faggioni, Alice Pomponi, Riccardo De Santis, Laura Masuelli, Andrea Ciammaruconi, Federica Monaco, Annapia Di Gennaro, Laura Marzocchella, Vittorio Sambri, Rossella Lelli, Giovanni Rezza, Roberto Bei, Florigio Lista

**Affiliations:** 1 Histology and Molecular Biology Section, Army Medical and Veterinary Research Center Via Santo Stefano Rotondo, 4 00184, Rome, Italy; 2 Department of Experimental Medicine, University of Rome “Sapienza”, Rome, Italy; 3 Istituto Zooprofilattico Sperimentale dell’Abruzzo e del Molise “G. Caporale”, Teramo, Italy; 4 Department of Clinical Sciences and Translational Medicine, University of Rome “Tor Vergata”, Rome, Italy; 5 Regional Reference Centre for Microbiological Emergencies (CRREM), Microbiology Unit, Azienda Ospedaliero-Universitaria di Bologna, Policlinico S. Orsola–Malpighi, Bologna, Italy; 6 Department of Infectious Diseases, Istituto Superiore di Sanità, Rome, Italy

**Keywords:** West Nile Virus, WNV, WARF4, N-NS4B/WARF4, Alternative open reading frame

## Abstract

**Background:**

West Nile Virus (WNV) is a flavivirus that requires an efficient humoral and cellular host response for the control of neuroinvasive infection. We previously reported the existence of six alternative open reading frame proteins in WNV genome, one of which entitled WARF4 is exclusively restricted to the lineage I of the virus. WARF4 is able to elicit antibodies in WNV infected horses; however, there was no direct experimental proof of the existence of this novel protein. The purpose of this study was to demonstrate the *in vitro* production of WARF4 protein following WNV infection of cultured VERO cells and its immunity in WNV infected individuals.

**Results:**

We produced a monoclonal antibody against WARF4 protein (MAb 3A12) which detected the novel protein in WNV lineage I-infected, cultured VERO cells while it did not react with WNV lineage II infected cells. MAb 3A12 specificity to WARF4 protein was confirmed by its reactivity to only one peptide among four analyzed that cover the full WARF4 amino acids sequence. In addition, WARF4 protein was expressed in the late phase of WNV lineage I infection. Western blotting and bioinformatics analyses strongly suggest that the protein could be translated by programmed −1 ribosomal frameshifting process. Since WARF4 is embedded in the NS4B gene, we rename this novel protein N-NS4B/WARF4. Furthermore, serological analysis shows that N-NS4B/WARF4 is able to elicit antibodies in WNV infected individuals.

**Conclusions:**

N-NS4B/WARF4 is the second Alternative Reading Frame (ARF) protein that has been demonstrated to be produced following WNV infection and might represent a novel tool for a better characterization of immune response in WNV infected individuals. Further serological as well as functional studies are required to characterize the function of the N-NS4B/WARF4 protein. Since the virus might actually make an extensive use of ARFs, it appears important to investigate the novel six ARF putative proteins of WNV.

## Background

West Nile virus (WNV) is an arthropod-borne virus maintained in a bird-mosquito transmission cycle. Birds are the natural reservoir hosts while humans and other mammals are dead-end hosts occasionally infected through mosquito bite [1]. The virus, which was identified in 1937 [2], has been the cause of sporadic cases and outbreaks of disease in Africa, Australasia, Europe, and Middle East [3-5]. Since 1996, WNV has gained growing importance in the western world, causing massive outbreaks and/or small clusters of encephalitis in Europe [6-8]. The virus was introduced for the first time in the North America in 1999 [9,10], spreading to several countries [11], and becoming a major public health problem in the USA [12].

WNV is a small enveloped virus [13] belonging to the *Flaviviriade* family, genus *Flavivirus*[14]. Phylogenetic analysis reveals two distinct viral lineages, lineage I and lineage II [15]. Lineage I is involved in human and equine outbreaks while lineage II is not associated with clinical manifestations in humans [15-17]. WNV genome is a positive single-stranded RNA of about 11 kb containing a single open reading frame flanked by two untranslated regions. RNA translation produces a long polyprotein processed by viral and cellular proteases in three structural (C, preM/M and E) and seven non structural proteins (NS1, NS2A, NS2B, NS3, NS4A, NS4B, NS5) [18]. Overall, the structural proteins are involved in virus binding and penetration of host cells [19], while the non-structural proteins are involved in the replicative cycle [20] and induce immunological evasion mainly through the inhibition of type I interferon signaling [21,22]. Still, it has been suggested that other unidentified factors could play a role in the pathogenesis of WNV neuroinvasive disease [23]. Melian et al. demonstrated that the NS1’ protein, a known variant of the canonical NS1 protein, results from a ribosomal frame shifting process [24]. The variant protein appears to play a role in WNV neuroinvasiveness. We recently reported the existence of six alternative open reading frames (ARFs) in the WNV genome by *in silico* analysis. We also demonstrated a significant antibody response to one of this six novel putative proteins (WARF4) in the serum of horses testing positive for antibodies to WNV. However, there was no direct experimental proof of the *in vivo* existence of this novel protein [25]. The aim of this study was to demonstrate that WARF4 protein is synthesized following WNV infection of mammalian cultured cells. To address this objective, a monoclonal antibody against WARF4 protein was produced. In addition, sera of WNV infected individuals were analyzed in order to test the capacity of WARF4 to induce an immune response in humans as well.

## Results

### Generation of a mouse monoclonal antibody against WARF4 protein

**Figure 1:**
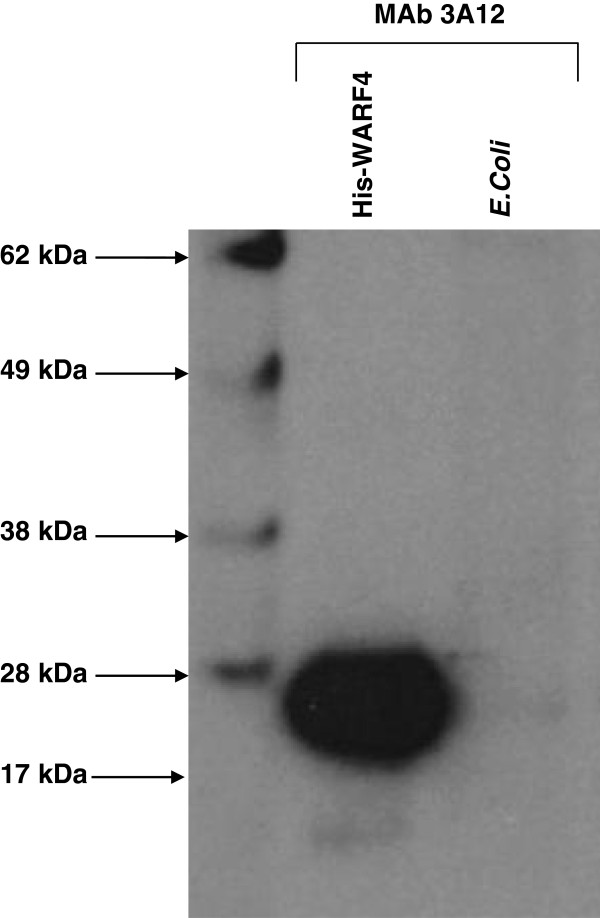
**Reactivity of MAb 3A12 with WARF4 recombinant protein.** Protein extracts from *E. coli* BL21 transformed with His-WARF4 and with the empty vector (pRSETC) were analyzed by western blotting. MAb 3A12 reacted with the recombinant His-WARF4 while it did not show reactivity with the crude lysate *of E. coli*.

In order to demonstrate the *in vitro* production of the WARF4 protein following WNV lineage I infection, a mouse monoclonal antibody to His-WARF4 fusion protein was generated. The selected MAb 3A12 recognized the His-WARF4 by western blotting while it did not show cross-reactivity with the crude lysate from *E. coli* transformed with the empty vector (Figure 1).

### In silico aminoacid alignment and identification of the N-NS4B/WARF4 region detected by MAb 3A12

**Figure 2:**
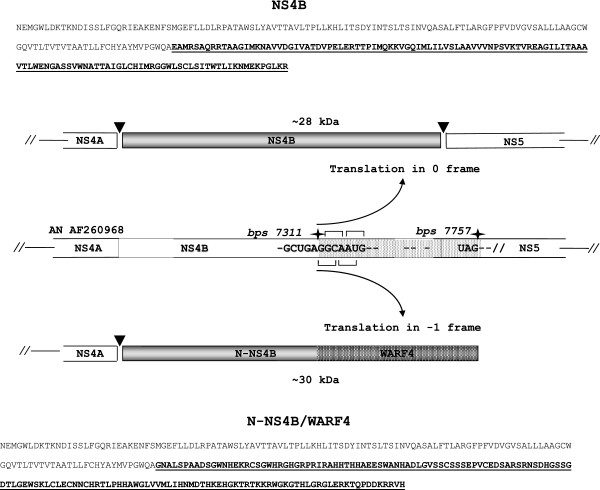
**Proposed mechanism of N-NS4B/WARF4 synthesis.** In the center the WNV 3^′^ genomic organization is shown. WARF4 is dashed, the first and the last base of the alternative reading frame are pointed by stars. On the top, the synthesis of NS4B by canonical translation mechanism in 0 frame and maturation is shown. Below is displayed the proposed mechanism of N-NS4B/WARF4 synthesis through translation in −1 frame. The amino acids sequences of the two proteins are showed, the different COOH terminals of the two proteins are underlined.

**Figure 3:**
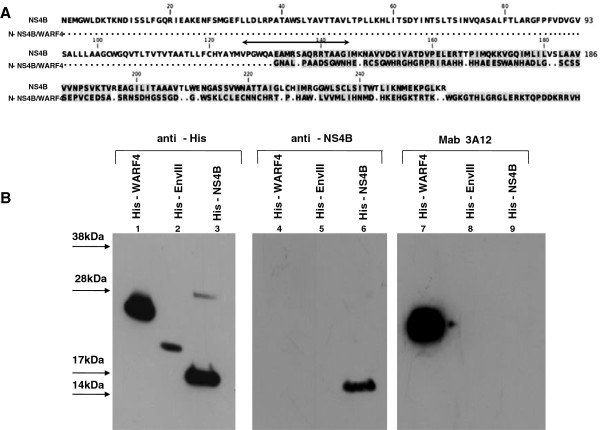
**(A) Comparative aminoacid sequence analysis.** Amino acids alignment of the NS4B and N-NS4B/WARF4. Identical residues are shown as dots. The arrow represents the 14 AA target of the anti-NS4B antibody. (**B**) Western blotting analysis of His-NS4B/His-WARF4 proteins employing MAb 3A12 and the anti-NS4B antibody. The analysis was carried out to demonstrate the different amino acids composition of N-NS4B/WARF4 protein relative to the terminal region of NS4B protein. The first three lanes show the reactivity of the anti-His antibody with the His-tagged proteins used in the same western blotting as positive controls. The anti-NS4B antibody did not react with the His-WARF4 protein (lane 4). Similarly, the MAb 3A12 did not react with His-NS4B protein (lane 9). The His-Envelope protein was used as negative control.

WARF4 is an alternative gene overlapping the COOH-terminal region of the NS4B gene and a small NH2-terminal portion of the NS5 gene (Figure 2). The genomic organization of WNV implies that a −1 ribosomal frameshifting process translates WARF4, thus the novel protein has been renamed N-NS4B/WARF4. The amino acid composition of N-NS4B/WARF4 is completely different from the NS4B viral protein in the COOH-terminal region, this is shown by the amino acid alignment analysis in Figure 3A. To further support the bioinformatics evidence, a western blotting analysis of the recombinant His-WARF4 and His-NS4B proteins employing the polyclonal anti-NS4B antibody and MAb 3A12 was performed to demonstrate that His-WARF4 and NS4B proteins are dissimilar (Figure 3B). The commercial polyclonal antibody anti-NS4B was developed to a NS4B fragment from AA 126 to AA 145, which thus overlaps the first 14 AA of the N-NS4B/WARF4 COOH-terminal region. As shown in Figure 3B (lane 4), the anti-NS4B antibody did not recognized the recombinant His-WARF4 protein. The recombinant His-NS4B fragment encompasses the COOH-terminal portion of NS4B starting from AA 120 thus including the entire ARF coding sequence. In addition, MAb 3A12 did not recognize the His-NS4B fragment (lane 9).

**Figure 4:**
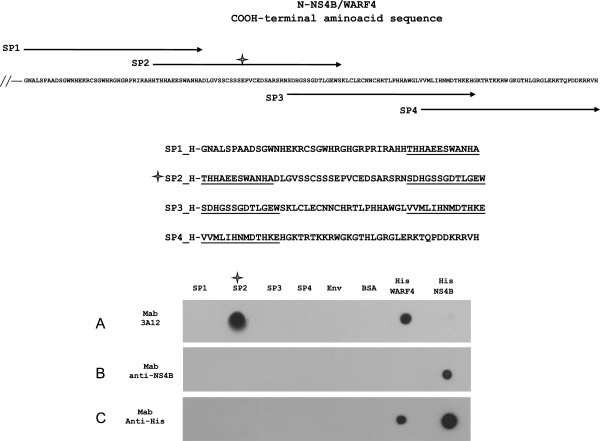
**Identification of the N-NS4B/WARF4 COOH-terminal amino acids sequence detected by MAb 3A12.** The amino acids sequence of the N-NS4B/WARF4 COOH-terminal region with the graphical representation of the four synthetic peptides (SP1, SP2, SP3, SP4) covering the full amino acids sequence codified by WARF4 is shown on the top. The amino acids sequence of the four synthetic peptides is showed in the center, the overlapping sequences among the contiguous peptides are underlined. Peptides (500 ng), Envelope protein and BSA (500 ng) and His-WARF4 and His-NS4B proteins (50 and 150 ng, respectively) were analyzed by dot blotting employing MAb 3A12 (panel **A**), anti-NS4B antibody (panel **B**) and MAb anti-His (panel **C**). The star shows the peptide (SP2) recognized by MAb 3A12.

The N-NS4B/WARF4 region recognized by MAb 3A12 was identified by analyzing MAb 3A12 reactivity to four synthetic overlapping peptides (SP1, SP2, SP3, SP4) which cover the full N-NS4B/WARF4 COOH-terminal amino acids sequence (Figure 4). The four synthetic peptides, as well as recombinant His-WARF4, His-NS4B, Env proteins and Bovine Serum Albumin (BSA) were spotted in replicates on nitrocellulose membranes. Membranes (panels A, B, C) were then reacted with MAb 3A12, anti-NS4B antibody and anti-His antibody respectively. As shown in Figure 4, MAb 3A12 recognized only SP2, while lacking reactivity to His-NS4B, His-EnV and BSA. Conversely, anti-NS4B or anti-His antibody did not recognize any of the WARF4 peptides. These results corroborate the specificity of MAb 3A12 to N-NS4B/WARF4 protein and allow to recognize the epitope of MAb 3A12 between amino acids 165–212 of N-NS4B/WARF4 sequence.

### Expression of N-NS4B/WARF4 protein in WNV lineage I infected cells

**Figure 5:**
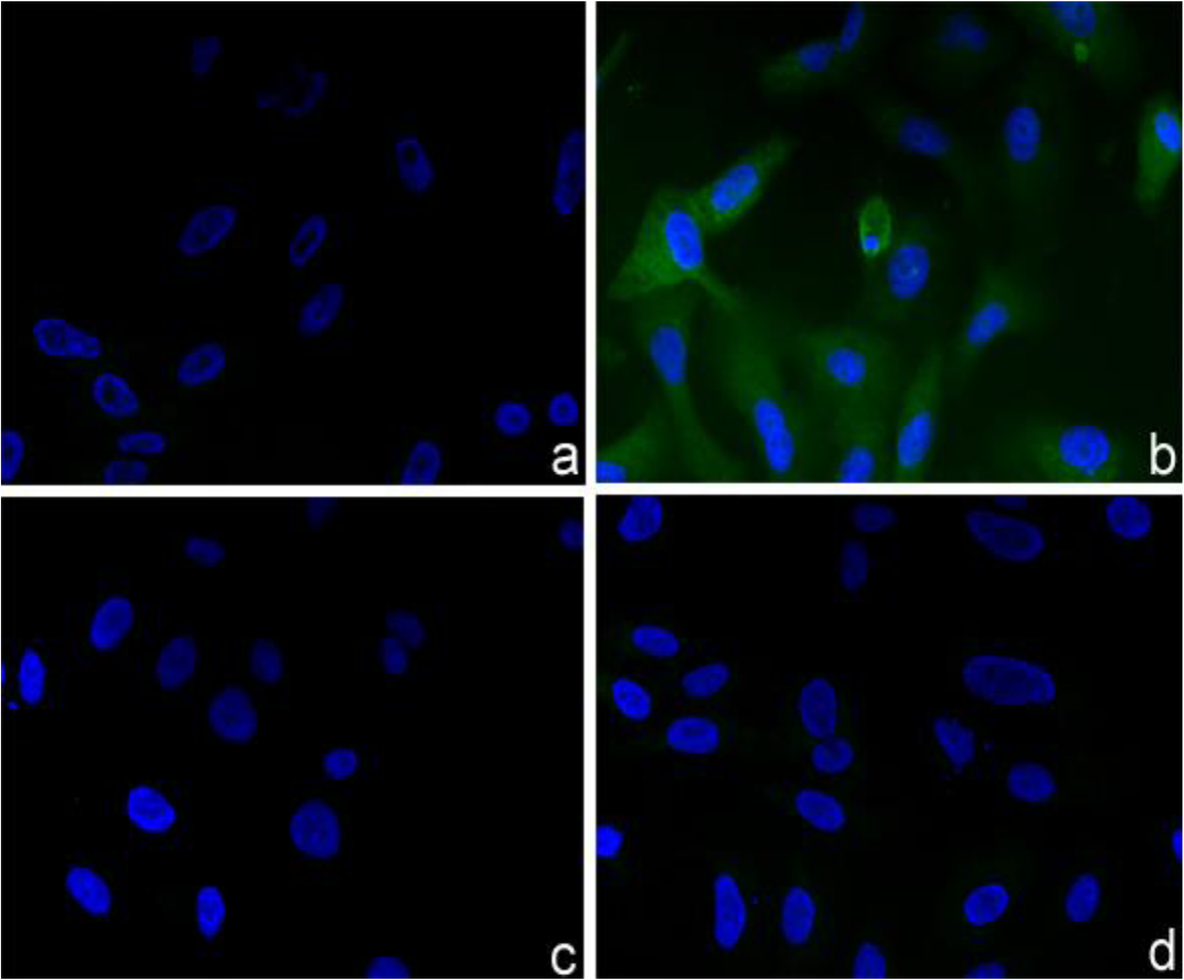
**Expression and intracellular localization of N-NS4B/WARF4 in VERO infected cells.** Reactivity of MAb 3A12 with the cytoplasm of VERO WNV infected cells (panel **b**). No reactivity was observed with non infected cells (panel **d**). MOPC-21 was used as negative control with infected (panel **a**) and non infected cells (panel **c**).

Expression of N-NS4B/WARF4 following WNV lineage I infection of VERO cells in vitro was demonstrated by immunofluorescence and western blotting analyses employing MAb 3A12. Figure 5 shows the reactivity of MAb 3A12 with uninfected and infected VERO cells. MAb 3A12 showed a strong cytoplasmic immunoreactivity in infected cells (panel b), while it did not react with uninfected cells (panel c). MOPC-21 was used as negative control (panel a and c).

**Figure 6:**
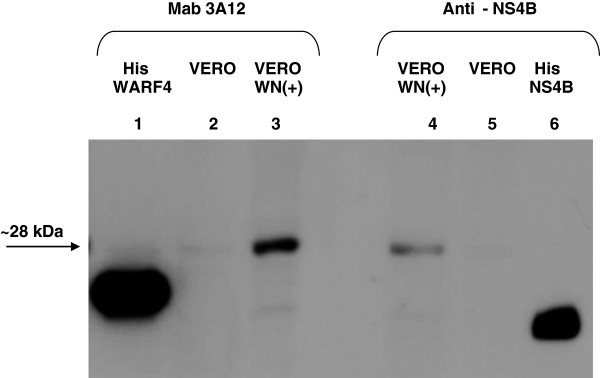
**Reactivity of MAb 3A12 with VERO WNV lineage I infected cells by western blotting.** MAb 3A12 detects a protein with an apparent molecular weight of about 28 kDa in WNV lineage I infected VERO cells (line 3), no reactivity was observed in uninfected VERO cells (lane 2). The recombinant His-WARF4 protein was used as positive control (lane 1). The commercial anti-NS4B antibody was used to monitor the infection of VERO cells and to compare the migration of NS4B protein (lane 4) with the novel protein. The electrophoretic mobility of N-NS4B/WARF4 protein (lane 3) resulted slightly less than NS4B protein (lane 4). The recombinant His-NS4B positive control was loaded with a delay of about twenty minutes (lane 6).

In order to determine the apparent molecular weight of the *in vitro* produced N-NS4B/WARF4 protein, western blotting analysis was carried out. As shown in Figure 6, in infected VERO cell lysate MAb 3A12 detected a protein showing an apparent molecular weight of about 28 kDa (lane 3). Recombinant His-WARF4 (lane 1) was used as positive control. No reactivity of MAb 3A12 was observed with uninfected VERO cells (lane2). The commercially available anti-NS4B antibody was used as positive control to monitor the infection of VERO cells by WNV (lane 4) and to compare the apparent molecular weight of NS4B protein to N-NS4B/WARF4 protein. Our result shows that the electrophoretic mobility of N-NS4B/WARF4 is slightly lower than that of NS4B. The recombinant His-NS4B was used as positive control for anti-NS4B antibody (lane 6).

**Figure 7:**
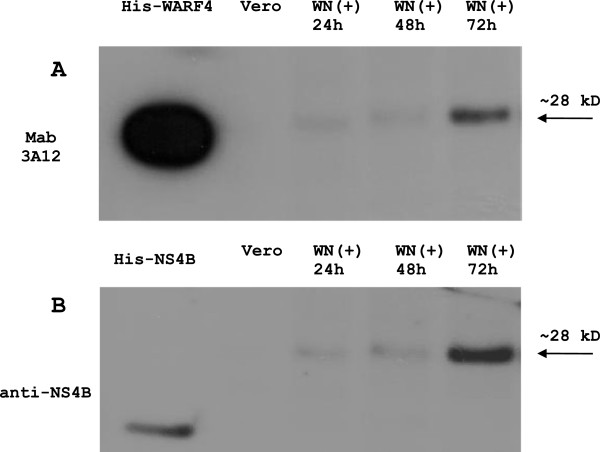
**Time-course of VERO cells infection and western blot analysis.** The expression of the N-NS4B/WARF4 protein (panel **A**) was evaluated by western blotting analysis and compared to the expression of the NS4B protein (panel **B**) at 24–72 hours post-infection. Both proteins show a similar behaviour with maximum expression in the late phase of infection. His-WARF4 (20 ng) and His-NS4B (50 ng) were used as positive controls.

Next, the expression of N-NS4B/WARF4 was evaluated and compared to the expression of NS4B protein through a time-course infection (24–72 hours). Figure 7 shows the results of western blotting analysis performed with MAb 3A12 (panel A) and polyclonal anti-NS4B (panel B). N-NS4B/WARF4 and NS4B proteins show similar kinetics of expression after WNV lineage I infection, the highest level of expression of both in the late phase of infection.

### N-NS4B/WARF4 expression is restricted to WNV lineage I

**Figure 8:**
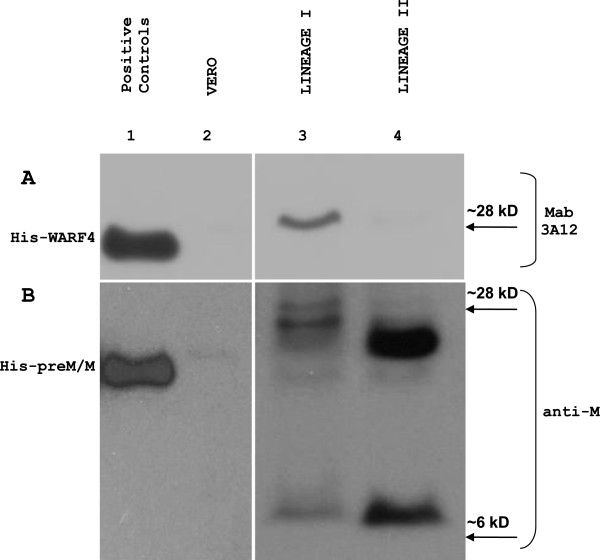
**N-NS4B/WARF4 expression is restricted to WNV lineage I.** Panel **A** shows the reactivity of WNV lineage I with MAb3A12 (lane 3), no reactivity was observed in WNV lineage II (lane 4), VERO cells were used as negative control (lane 2). To monitor the infection of VERO cells with both lineage I and II, the membrane was reprobed with a commercial antibody anti-M (panel **B**). The M protein and other immature forms were detected in both the WNV lineage I and II (lanes 3 and 4). His-WARF4 and His-preM/M proteins were used as positive controls.

To corroborate bioinformatic analysis indicating that N-NS4B/WARF4 is restricted to WNV lineage I, the reactivity of MAb 3A12 was analyzed against WNV lineage II infected VERO cells (Figure 8). Infection of VERO cells was performed for 72 hours with WNV lineage I and lineage II. Western blotting analysis shows that MAb 3A12 identifies N-NS4B/WARF4 protein in the WNV lineage I infected VERO cells (panel A, line 3) but not in WNV lineage II infected VERO cells (panel A, line 4). To monitor infection of VERO cells by WNV lineage I and II, infected cells were also analyzed for the expression of matrix protein (M) employing the anti-M antibody (panel B). The anti-M antibody recognized the mature form of M protein showing a molecular weight of about 8 kD in both lineage I and II WNV infected cells (line 3 and 4). The antibody also detected an additional band representing the immature form of the M protein (preM/M). Recombinant His-WARF4 and His-PreM/M proteins were used as positive controls.

### N-NS4B/WARF4 induces antibodies in WNV infected individuals

**Figure 9:**
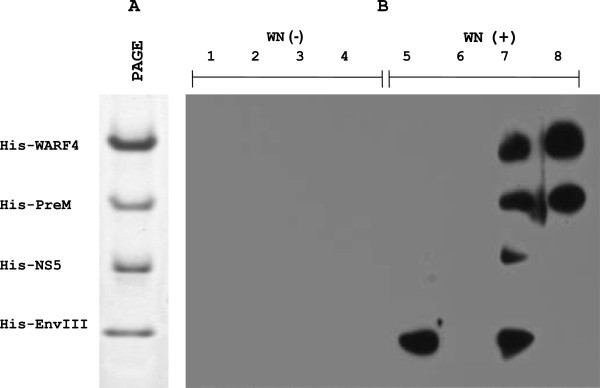
**Immune recognition of His-WARF4 by WNV-positive human sera.** Eight human sera, 4 of which positive for IgGs anti-WNV by IFA were analyzed for the presence of anti-N-NS4B/WARF4 antibodies by western blotting. The assay was performed by testing simultaneously the reactivity to 3 other recombinant WNV proteins: the domain III of the Envelope, a prem/M protein fragment and the NH_2_-terminal portion of NS5. Panel **A** shows the 4 recombinant proteins stained with the comassie-blue. Panel **B** shows the results of the western blotting analysis; 4 human sera testing negative for IgGs-anti WNV (1–4) showed no reactivity with the four antigens. The WNV-positive human sera (5–8) showed a different reactivity with the four recombinant antigens. Two sera (7, 8) reacted with His-WARF4 protein.

In order to determine whether human sera from WNV infected individuals were able to recognize N-NS4B/WARF4 protein, western blotting analysis was carried out. Reactivity of human sera to His-WARF4 protein was compared to that of 3 other recombinant WNV proteins, including the domain III of the envelope, a preM/M fragment and the NH_2_-terminal portion of NS5 (Figure 9A). Eight human sera, 4 of which positive for IgGs anti-WNV, were assayed. Sera from individuals testing negative for WNV by IFA and seroneutralization test were also negative for reactivity with recombinant WNV proteins. Conversely, sera from individuals testing positive for WNV by IFA and seroneutralization test showed different patterns of reactivity with the recombinant proteins analyzed (Figure 9B). Two sera were able to react with His-WARF4. Of these, one was able to recognize also the His-preM/M, His-NS5 and His-EnvIII proteins while the other recognized only the preM/M. In addition, one serum was able to detected EnvIII only, while another produced a weakly positive signal for the envelope and NS5 only after a long exposure (data not shown).

### Bioinformatic results

**Figure 10:**
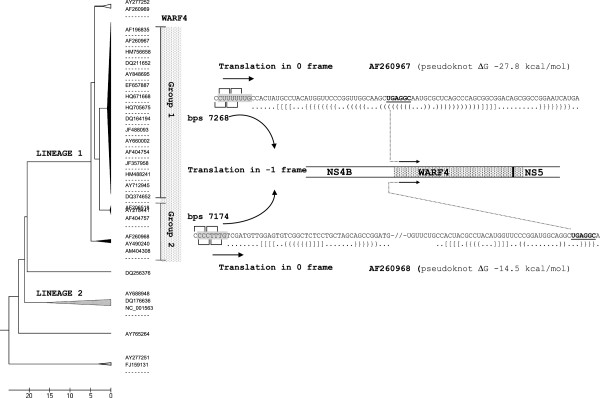
**UPMGA analysis of WNV genomes.** The image summarizes the results of the bioinformatics analysis. Some reference strains are shown. Sequence alignment identifies WARF4 in 98% of strain belonging to lineage I (black collapsed form). The WARF4 group (dashed) may be further separated in two groups, depending on the type of programmed −1 ribosomal frame shifting sequence detected. Group 1 carries the UUUUUUG slyppery sequence. Group 2 carries the CCCUUUG/T slippery sequence. The two pseudoknot sequences with the predicted base pairing are shown. The slippery sequences are in gray while the stop codon (AUG) and the first codon (GGC) of WARF4 are underlineed.

Alignment and cluster analysis of 384 WNV strains assigned 368 samples to lineage I according to previous studies [15] (Figure 10, Table 1). WARF4 was found in 361 genomes out of 368 belonging to lineage I. Two different mutually exclusive slippery sequences within the NS4B gene were detected in the WARF4 group. The first slippery sequence (UUUUUUG), with a pseudknot structure of 80 nucleotides (ΔG −27.8 kcal/mol) [bps 7268, an. AF260967] positioned 6 nucleotides downstream, is shared by 91% of genomes. The second slippery sequence (CCCUUUG/T), with a downstream pseudknot structure of 40 nucleotides (ΔG −14.5 kcal/mol) [bps 7174, an. AF260968] positioned 8 nucleotides downstream, is shared by 5% of genomes. The WARF4 sub-group genomes lacking slippery sequences are 4%.

**Table 1:** WNV accession numbers

										
**LINEAGE I**	**WARF4 Group 1**	AY646354	DQ066423	HQ891010	DQ164186	JF730043	DQ164199	HM488224	HM488159	AY660002
		HM756658	FJ411043	HQ891011	HM488183	DQ164198	HM488208	HM488172	AY289214	JF488087
		HM488118	HQ671697	HQ891013	HM488142	DQ164205	HM488253	HQ671702	HM488194	HM488218
		HM488119	HQ671714	JF730040	HM488147	HM488177	HM488133	HM488180	HM756660	HM488236
		HM488123	HM488247	HQ671669	DQ164200	HM488251	HM488136	HM488189	HM488165	HM756678
		HM488124	HM756661	HQ671673	HM488181	HM488140	HQ671719	DQ005530	HM488199	HM488206
		JF488089	HM756663	HQ671677	HM488209	HM756672	HQ671713	HM488162	HM488243	HM488238
		HM488237	HQ671711	HQ671687	HQ671722	HM488141	AF404755	HM488195	JF488097	HM488203
		DQ666452	AF404756	HQ671688	HM756667	HQ705659	GQ379156	FJ527738	HM488205	GQ379157
		HQ671715	EF657887	HQ671689	HQ671723	HM488114	DQ164202	HQ671701	HM488171	GQ379158
		HM488200	EF530047	HQ671690	HM488137	HM488139	AY712947	HM488227	HM488230	HM488233
		HQ671672	HM756662	HQ705670	HQ671699	HQ671724	DQ164189	HM488228	HQ671726	HM488234
		HQ891012	HM756664	HQ705674	HM488138	HM756650	EU155484	HM488176	HM488201	HM488116
		HQ671718	HM488130	HQ671682	HM488211	HQ705660	HM488231	HM488163	HM488207	JF488086
		HQ671730	HM488249	HQ671683	HM756673	HM488214	HM488232	HM488164	HM488245	HM756657
		HQ671728	HM488132	HQ671685	DQ164191	HM488216	HM488121	HM488161	HM488202	GQ379160
		JF488096	HQ671708	HQ705675	HM756648	HM488145	HM488122	HM488246	HM488179	JF488088
		HM488235	HQ671710	JF357959	AY795965	HM488146	HQ671703	HM488167	JF357960	HQ671671
		HQ671721	AF404753	HQ705672	HM488156	HM488144	HM488155	HM488170	HQ705669	JF357958
		HM488127	HQ671712	HQ671681	HM488158	HM488157	HQ671720	HM488168	HM756676	JF784158
		HM488128	HQ671668	HM488248	HM488148	HM756671	DQ164206	HM488169	HQ671698	HQ671676
		AF196835	HQ671680	DQ164188	HM488151	HQ671729	HM488175	HM488166	HM756669	DQ118127
		HM488125	HQ671674	HM488134	HM488154	HM488225	HM756653	HM488174	HM488193	DQ374652
		HM488126	HQ671675	HM488135	HM488150	HM488226	DQ164190	HM488204	HQ671700	DQ411029
		AF260967	HQ671679	HQ671716	HM488153	HM488229	HQ671725	HM488254	HM488217	
		HQ596519	HQ671684	HQ671717	HM488115	HM488185	HM488191	HM488196	HQ671692	
		DQ211652	HQ671678	HQ671696	HM488117	HM488143	HM488198	HQ671727	HQ671707	
		NC_009942	HQ671686	DQ164194	HM488120	HM488221	HM488173	HM756675	HQ671709	
		AY842931	HQ671691	DQ164193	HM488149	DQ164196	HM756659	HM488244	HM488240	
		EF571854	HQ671732	HM488178	HM488152	DQ164197	HM488210	HQ671705	HM756666	
		AY848695	HQ671733	HQ671693	HM756656	AY712948	HM488197	HM488188	JF730042	
		AY848696	HQ671731	HQ705677	HM488213	HM488186	HM756652	HM488219	HM488239	
		HM488131	HQ705663	HQ671694	JF488091	HM488190	HM756654	AF481864	HQ671704	
		AF404754	HQ705671	HQ705678	HM488215	HM488222	DQ164204	HM488252	HM488160	
		HM488129	HQ705673	HQ671695	JF488094	HM756665	DQ176637	JF488093	HM756649	
		AF206518	HQ671670	HM488250	HM488220	HM756668	AY712945	JF488090	HM488242	
		HQ671706	HQ705676	HM488182	HM756670	HM488212	AY712946	JF488092	HM488241	
		AY848697	HQ891009	DQ164195	HM488184	HM756651	HM488223	DQ666450	JF488095	
	**WARF4 Group 2**	DQ411034	DQ411035	DQ374651	DQ377179	DQ377178	DQ377180	DQ411033	DQ411032	DQ374650
		AY278441	DQ411030	DQ411031	AF404757	AF260968	EU081844	AM404308	EU249803	HM051416
		AY490240	AY603654							
		HM488187	DQ164187	DQ164192	DQ164203	DQ164201	DQ666448	DQ666451	HM756677	
		DQ666449	GQ379159	AY277252						

The table shows the accession numbers of WNV strains belonging to lineage I analyzed *in silico* in this work.

## Discussion

The use of ARFs in viruses belonging to the *Flaviviriade* family was first reported for the hepatitis C viruses [26,27]. Recently, it has been demonstrated that WNV uses a short ARF, termed *foo*, for the synthesis of NS1’ , a known variant of the canonical NS1 [24,28]. We earlier reported the presence of other ARFs embedded in the coding frame of the WNV genome [25]. Our bioinformatic analysis detected six ARFs, one of which, designed WARF4, was the longest and restricted exclusively to lineage I of WNV. Since WARF4 is embedded in the NS4B gene, the novel protein has been renamed N-NS4B/WARF4. Our results suggested the production of N-NS4B/WARF4 protein in WNV infected horses because of their ability to mount a humoral immune response to N-NS4B/WARF4. However, there was no direct evidence proving the actual existence of the N-NS4B/WARF4 protein. In order to demonstrate the production of N-NS4B/WARF4 *in vitro* after WNV cells infection, we produced a monoclonal antibody to the N-NS4B/WARF4 COOH-terminal amino acid sequence (MAb 3A12). MAb 3A12 strongly reacted with VERO WNV infected cells by immunofluorescence and detected a ~ 28-kDa protein by western blotting. The predicted aminoacids of N-NS4B/WARF4 preclude the possibility that MAb 3A12 could react with epitopes shared with the NS4B protein (Figure 3A), however to support this prediction a western blotting analysis was performed. As shown in Figure 3B, MAb 3A12 did not recognize the recombinant COOH terminal portion of the recombinant his-tagged NS4B protein. In addition, the anti-NS4B did not recognize the recombinant His-tagged WARF4 protein. To definitely asses the specificity of MAb 3A12 against the alternative reading frame, four overlapping peptides covering the full N-NS4B/WARF4 COOH- terminal amino acid sequence were synthesized and analyzed for their reactivity to MAb 3A12. As shown in Figure 4 the results confirm the specificity of the monoclonal antibody and allow to recognize its epitope between the amino acids 165–212 of N-NS4B/WARF4 sequence. In addition we demonstrated that N-NS4B/WARF4 protein expression is restricted to WNV lineage I infection and that it is expressed at high level in the late phase of infection (Figures 7, 8). Overall, our results demonstrate that N-NS4B/WARF4 is a novel protein, different from NS4B, and that is expressed in WNV infected cells.

Furthermore, we indirectly demonstrated the “*in* vivo” production of N-NS4B/WARF4 by showing its immunoreactivity with human sera obtained from WNV infected patients (Figure 9). The heterogeneous reactivity to the recombinant WNV antigens displayed by sera testing positive for WNV reflects the complex humoral response elicited by WNV infection [29,30]. In addition, it is known that the ARF proteins are expressed with both less and variable efficiency if compared to the canonical proteins [31].

To date, we have no experimental information on N-NS4B/WARF4 protein translation, but it appears reasonable to assume that a −1 ribosomal frame shifting mechanism produces the novel protein. Indeed, in *Flaviviriade* the translation process is implemented by a cap-dependent scanning process, which produces a single polyprotein [32]. The sequence encoding N-NS4B/WARF4 COOH-terminal is in −1 frame, moreover it is far from the 5^′^ terminal end, lacks an AUG codon and no internal ribosomal entry site (IRES) is described for WNV. The translation by ribosomal frame shifting is the only realistic explanation for N-NS4B/WARF4 protein synthesis. Since the proposed model requires the presence of specific RNA structures such as slippery sequences associated with pseudknot [33-35], a bioinformatics analysis was performed to predict these structures. All the complete genomes of WNV available on gene bank were aligned and assigned to the two main lineages (Figure 10, Table 1). The strains belonging to the lineage 1 were first analyzed to confirm the association with WARF4 and then a further analysis was carried out looking for slippery sequences and pseudknot structure within the NS4B coding region. WARF4 was detected in 361 out of 368 genomes belonging to lineage 1. Seven genomes lacked WARF4 because of a single nucleotide substitution that interrupts the alternative frame. In the WARF4 group, two different and mutually exclusive slippery sequences with downstream frameshift-stimulating pseudknot structures were predicted. The first UUUUUUG sequence is the most representative (91%). The pseudknot structure is 80 nucleotides long and includes the initial aminoacids codified by the −1 frame. This slippery sequence is associated with the American viral strains. The second CCCUUUG/T sequence is present in 5% of the genomes, it is positioned 129 nucleotide upstream of WARF4 and has a pseudoknot structure of 40 nucleotides. The second slippery sequence is associated with circulating viral strain in Mediteranean Bacin and Est-Europe. The strain analyzed in this work belongs to this second group [Egyptian strain, an. AF260968]. It should be noticed that both the structures must promote the suppression of a termination codon (UGA) located just before the first codon of −1 frame [36].

The ribosomal frameshifting model also explains the discrepancy between the predicted and the observed molecular weight of the alternative N-NS4B/WARF4. The predicted alternative reading frame protein consists of 148 AA that account for a molecular weight of 16,7 kDa. However, the protein detected by MAb 3A12 in WNV infected cells migrates with an apparent molecular weight of about 28–30 kDa. Although this discrepancy could be due to a post-translation modification, the proposed model appears the most reasonable explanation for the observed molecular weight of N-NS4B/WARF4 protein. The novel protein would exist as COOH-terminal variant of the NS4B protein, indeed, the ribosomal shift in −1 frame would give rise to a NS4B variant protein where the last 123 AA should be replaced by a longer amino acid tail of 148 A. Thus, the variant protein should exhibit a molecular weight of about 30 kDa, consistent with our results (Figure 6, lane 3). The proposed ribosomal frameshifting model implies that the expression kinetic of N-NS4B/WARF4 should be like that of NS4B protein even if the amount of the novel protein should be less than that of NS4B. Figure 7 shows the expression of N-NS4B/WARF4 and NS4B in time-corse infection, the two proteins exhibit a similar kinetics. N-NS4B/WARF4 is clearly detected in the late phase of infection, such as the NS4B protein. The level of NS4B and N-NS4B/WARF4 proteins expression based on a densitometric analysis (data not shown) indicates a ratio of about 25 to 1 respectively. It should be highlighted that this ratio is estimated for the strain Eg101 [an. AF260968] that our biofinformatic analysis associates with the strain circulating in the Mediterranea area (Figure 10). The predicted pseudoknot associated with the American strain is thermodinamically more stable. It should be intersting to estimate this ratio in the American viral strain.

## Conclusions

Overall, our results show for the first time that the novel ARF protein, N-NS4B/WARF4, is produced during the late stage of WNV lineage I infection and that N-NS4B/WARF4 is able to elicit antibodies in WNV infected individuals. To date, the biological function of N-NS4B/WARF4 and the role of anti- NS4B/WARF4 antibodies are unknown; however, it is suggestive that N-NS4B/WARF4 is restricted exclusively to the lineage I of WNV, which is known to be associated with the more severe clinical manifestations of WNV disease. This protein might represent a novel tool for a better understanding of WNV biology and for an improved characterization of immune response in WNV infected individuals.

## Methods

### Cell culture and virus strain

The VERO E6 cell line was cultured in Eagle’s minimal essential medium (MEM) with 10% Fetal Calf Serum (FCS), 100 U/mL penicillin, 200 μg/mL streptomycin in the presence of 5% CO2. WNV lineage I [strain Eg101, an. AF260968] and WNV lineage II [strain B956, an. AY532665] were propagated by infecting VERO E6 cells monolayers in MEM with 2% FCS and titrated according to the Reed and Muench formula.

Additional WNV genomic RNA [strain NY 1999, an. AF260967] was obtained from European Network for Diagnostics of Imported Viral Disease (http://www.enivd.de/ENIVD_P.HTM) during the External Quality Assurance (EQA) for molecular detection of West Nile virus. The nucleic acids were extracted by NucleoSpin RNA Virus kit according to manufacturer’s instructions (Macherey-Nagel, Düren, Germany).

### Expression of WARF4, NS4, envelope, preM/M and NS5 recombinant proteins

Since the previously described recombinant His-WARF4 protein [25] does not comprise the full alternative reading frame, we cloned a novel fragment which includes the entire ARF. The 444 bps fragment spanning the WARF4 position 7311–7754 [accession number AF260967] was amplified in a single step by Superscript III One step RT-PCR, (Invitrogen, California). The fragment was cloned in pRSET vector and expressed in the BL21 Star (DE3)pLysS competent cells (Invitrogen, California). Other WNV recombinant proteins including the domain III of the Envelope (Env III), a preM/M and a NS5 fragment were produced as described above using primers and vectors reported in Table 2. The NS4B fragment was expressed by Rapid Translation System 100 E.coli HY (http://www.5PRIME.com). The His-tagged recombinant proteins were purified by Ni-NTA affinity chromatography kit according to manufacturer’s instructions (QIAGEN, Hilden, Germany). All the oligonucleotides were synthesized by Eurofins MWG Operon (http://www.eurofinsdna.com). 

**Table 2:** Primers and vectors used to generate WNV recombinant proteins

**Name**	**Position AF260967**	**Primer forward**	**Primer reverse**	**Vector**
WARF4	7310-7757	5^′^-atggcggatccaggcaatgcgctcagcccagcg-3^′^ BamHI	5^′^- tctttgaagcttctagtgaactcttcttttgtc-3^′^ HindIII	pRSET B
ENV	1852-2198	5^′^- ggaaaggatccagttgaagggaacaacc -3^′^ BamHI	5^′^-ctcctgaattcgtggttgtaaaggctttgcc-3^′^ EcoRI	pRSET C
preM/M	464-968	5^′^-cagcgggatcccagttaccctctctaacttcc-3^′^ BamHI	5^′^-caagggaattcaagctgtaagctggggccacc-3^′^ EcoRI	pRSET C
Partial NS5	9015-9645	5^′^-gaggcggatccgcggggggaatgtcacac-3^′^ BamHI	5^′^-ttccgaattctcaaacagccaggtcctg-3^′^EcoRI	pRSET B
Partial NS4B	7276-7680	5^′^-tcgatggatccgacactatgcctacatggttcc-3^′^BamHI	5^′^-ggatcaagcttttatctttttagtcctggtttttc-3^′^HindIII	pRSET C

The table shows the primers and vectors used for molecular cloning. The restriction sites are underlined. Because of BamHI restriction site generated by a T/C transition in position 850 [AF260967], the preM/M cloned fragment encompassed the positions 464–850.

### Production of a monoclonal antibody (MAb) recognizing the WARF4 recombinant protein

Four week-old BALB/mice were immunized twice by intraperitonal injection with 25 μg of purified His-WARF4 protein emulsified in RIBI adjuvant (RBI Immununochemical Research). Mice were then given a booster immunization intravenously with 10 μg of the immunogen, and immune splenocytes were removed 3 days later. Somatic cell hybrids were prepared with NS-1 mouse non secreting myeloma cells as previously described [37-39]. Hybridoma supernatants were screened for differential immunoreactivity to His-WARF4 and His-purified control proteins by enzyme linked immunosorbent assay [40,41]. Positive hybridoma cell lines were cloned twice by limiting dilution. One MAb was selected and designed 3A12.

### Peptide scanning analysis

Four synthetic peptides named SP1, SP2, SP3 and SP4, which cover the full N-NS4B/WARF4 COOH-terminal amino acid sequence and which have amino acids overlapping sequences were designed (Figure 4).

SP1 (H-GNALSPAADSGWNHEKRCSGWHRGHGRPRIRAHHTHHAEESWANHA), SP2 (H-THHAEESWANHADLGVSSCSSSEPVCEDSARSRNSDHGSSGDTLGEW), SP3 (H-SDHGSSGDTLGEWSKLCLECNNCHRTLPHHAWGLVVMLIHNMDTHKE) and SP4 (HVVMLIHNMDTHKEHGKTRTKKRWGKGTHLGRGLERKTQPDDKRRVH) were synthesized by PEPSCAN (http://www.pepscan.com). Five hundred ng of peptide as well as Env and BSA proteins, 50 ng of His-WARF4 and 200 ng of His-NS4B were spotted in replicates on nitrocellulose membranes. Membranes were blocked for 4 h at RT in 5% non-fat dry milk-PBS-0.1% Tween-20 and incubated 1 hour at RT with MAb 3A12, anti-NS4B antibody (Abcam plc, Cambridge, UK) and MAb anti-Histidine (Invitrogen). The membranes were washed four times and then incubated with goat anti-mouse or rabbit IgG peroxidase-conjugated antibodies (Sigma, MI, IT) and developed by a chemiluminescent kit (Sigma, MI, IT) as previously described [42,43].

### Indirect immunofluorescence assay (IFA)

The VERO E6 cell line was grown in eight wells Chamber slides™ (Nunc, USA). 200 μl of a viral suspension (10^4^ xTCID50/ml) were used to infect VERO cells monolayers (40-50% confluent). WNV was subsequently allowed to adsorb for 1 hour at 37°C. MEM medium with 2% FCS, was then added to the infected cells monolayer. After 36 hours, the cells monolayer was washed 2 times in PBS 1X and fixed with 4% paraformaldehyde for 20 min at room temperature, followed by treatment with 0.1 M glycine for 20 min at 25°C and with 0.1% Triton X-100 for an additional 5 min at 25°C to allow permeabilization. Cells were incubated for 30 min at room temperature with MAb 3A12 or the antibody MOPC-21 used as negative control as previously described [44,45]. Nuclei were stained with Hoechst 33342 (blue).

### Western blotting analysis

The VERO cells were infected with a multiplicity of infection (MOI) of 0.1 for 24 to 72 hours or for 96 hours at 37°C in 5% CO2. Cells were scraped and harvested by centrifugation at 1,500 X g at 4°C and rinsed in ice-cold phosphate-buffered saline (PBS). Cell lysates were prepared in Staph A buffer (10 mM sodium phosphate pH 7.4, 100 mM NaCl, 5 mM EGTA, 1% Triton, 0.1% SDS, 0.5% deoxycholate) containing 1 mM PMSF [46,47] and a protease inhibitor cocktail (complete Mini EDTA-free, Roche). Proteins from uninfected and infected VERO cells lysate (50 μg/well) or purified recombinant WARF4 (10–100 ng/well), Env III (10–100 ng/well), preM (100 ng/well), NS5 (100 ng/well), NS4B (10 ng/well) protein fragments were separated on a NuPAGE 4-12% or 12% Bis-Tris gel and transferred onto nitrocellulose membranes (Invitrogen) [48]. Membranes were blocked for 6 h at 4°C in 5% nonfat dry milk-PBS-0.1% Tween-20 and incubated overnight at 4°C with MAb 3A12, anti-NS4b antibody, anti-M (Abcam plc, Cambridge, UK) or human sera (dilution 1:100). The membranes were washed four times and then incubated with goat anti-mouse IgG, anti-rabbit or anti-human IgG peroxidase-conjugated antibodies (Sigma, MI, IT) and developed by a chemiluminescent kit (Sigma, MI, IT) as previously described [42,49].

### Human sera

Human serum samples, obtained from convalescent patients suffering from neuro-invasive WNV infection, testing positive for IgGs anti-West Nile by IFA and confirmed by Micro-Neutralization Test Assay - MNTA [50] were kindly provided by Dr. Vittorio Sambri (St. Orsola-Malpighi University Hospital, University of Bologna, Bologna, Italy). Human sera from healthy donors testing negative for IgGs anti-West Nile were used as negative controls.

### Bioinformatic analysis

384 full genomes of WNV were retrived from gene bank and aligned with BioNumerics software package (version 6.5 Applied-Maths, Belgium). Genomes were further analized by KnotInFrame software (http://bibiserv.techfak.uni-bielefeld.de/knotinframe/) to predict ribosomal −1 frameshift sites with a pseudoknot strucuture.

### Human sera and experimental animals

Human sera were collected after informed consent and sent to the Regional Reference Centre for Microbiological Emergencies (CRREM, Prof. Vittorio Sambri, St. Orsola-Malpighi University Hospital, University of Bologna, Bologna, Italy). The protocol was approved under the regional plan of surveillance and control for west nile disease in Emilia Romagna (5 June 2009, prot. PG/2009/128190).

Mice were sacrificed by cervical dislocation and later the spleens were removed by surgical resection. The procedures were carried out in the presence of a veterinarian who monitored proper compliance with the decree law 116/92. The protocol was approved by the ethics committee of the Station Technology for Animal (STA) (http://www.sta.uniroma2.it/) of the University of Rome “Tor Vergata” on 06/12/2010.

## Abbreviations

WARF4: West Nile Alternative open Reading Frame 4; WNV: West Nile Virus; ARF: Alternative Reading Frame.

## Competing interests

The authors declare that they have no competing interests.

## Authors’ contributions

GF, RB, , and FL participated in the design of the study, carried out the experiments and wrote the manuscript. AP, RDS, LM, AC and LM carried out the experiments and wrote the manuscript. GR, FM, RL, ADG and VS have critically revised the manuscript and the experimental design. All authors read and approved the final manuscript.
